# Exploring the gut microbiome in type 2 diabetes across different insulin resistance levels: a machine learning approach

**DOI:** 10.3389/fnut.2026.1747767

**Published:** 2026-01-28

**Authors:** Yuchi He, Lu Liu, Yifan Liu, Jialong Jia, Yuqing Chen, Xiyu Zhang, Ya Liu

**Affiliations:** 1 School of Clinical Medicine, Chengdu University of Traditional Chinese Medicine, Chengdu, China; 2 Institute of Traditional Chinese Medicine, Sichuan Academy of Chinese Medicine Sciences (Sichuan Second Traditional Chinese Medicine Hospital), Chengdu, China; 3 Department of Endocrinology, Traditional Chinese Medicine Hospital of Meishan, Chengdu, China; 4 Department of Endocrinology, Hospital of Chengdu University of Traditional Chinese Medicine, Chengdu, Sichuan, China

**Keywords:** gut microbiome, insulin resistance, machine learning, type 2 diabetes mellitus, XGBoost

## Abstract

**Introduction:**

Insulin resistance (IR) is central to type 2 diabetes mellitus (T2DM). Composite indices including the atherogenic index of plasma (AIP), metabolic score for insulin resistance (METS-IR), triglyceride–glucose index (TyG), and TyG-BMI, are widely used to quantify IR severity. The gut microbiome (GM) has been implicated in metabolic dysregulation, but its associations with IR remain incompletely defined.

**Methods:**

We collected blood test results and stool samples from participants with T2DM and healthy controls. Stool samples underwent 16S rRNA gene sequencing. We trained XGBoost models to distinguish individuals with higher IR from healthy controls based on GM profiles and performed correlation analyses between GM features, clinical measures, and IR indices.

**Results:**

Triglycerides (TG), fasting blood glucose (FBG), and high-density lipoprotein cholesterol (HDL-C) differed significantly between the T2DM and control groups. IR indices (AIP, METS-IR, TyG, and TyG-BMI) were markedly higher in the T2DM group. XGBoost models based on GM profiles showed high discriminatory performance for identifying T2DM individuals with higher IR, with Bacteroides and Faecalibacterium contributing most to model performance. Correlation analyses further indicated that Lachnospiraceae_UCG-010, Bacteroides, Faecalibacterium, Lachnospira, Parasutterella, and Escherichia–Shigella were associated with clinical measures and IR indices.

**Conclusions:**

Specific GM features are associated with IR-related clinical measures and composite indices in T2DM, supporting their potential as intervention targets to improve insulin resistance and restore carbohydrate and lipid metabolism.

## 1 Introduction

Type 2 diabetes mellitus (T2DM) is a chronic metabolic condition characterized by dysregulation of carbohydrate metabolism, imposing an increasing burden to the global population (1). According to the International Diabetes Federation (IDF), approximately 536.6 million individuals aged 20–79 years were living with diabetes worldwide in 2021, and this number is expected to rise to 783.2 million by 2045 (2). Among the various types of diabetes, T2DM is the predominant form, with insulin resistance (IR) as its core pathophysiological feature.

IR refers to a condition in which insulin-sensitive tissues, especially the liver, muscle, and adipose tissue, are unable to effectively lower blood glucose levels in response to normal insulin concentrations (3). Although the level of IR cannot be measured directly in clinical practice, several indices have been developed to estimate IR status from different physiological perspectives. The atherogenic index of plasma (AIP), calculated from triglyceride (TG) and high-density lipoprotein cholesterol (HDL-C), reflects the balance between adverse and protective lipoproteins (4). Notably, a non-linear relationship between AIP and IR has been reported, with a positive correlation below an inflection point of 0.45 (5). The metabolic score for insulin resistance (METS-IR) is another index for quantifying IR, positively associated with visceral fat accumulation and fasting insulin levels, and has demonstrated superior performance in evaluating insulin sensitivity (6). The triglyceride-glucose index (TyG), derived from fasting blood glucose and triglycerides, has shown high sensitivity and specificity for diagnosing IR when benchmarked against euglycemic-hyperinsulinemic clamp, the gold standard for IR assessment (6). An extended version, TyG-BMI, incorporates body mass index (BMI) into the TyG index and exhibits a strong correlation with IR (7). Collectively, these indices enable accessible and cost-effective evaluation of IR, which is central to the development and progression of T2DM as well as its chronic complications. Consequently, strategies that ameliorate IR are critical for improving glycemic control and preventing T2DM-related morbidity.

Growing evidence indicates that the gut microbiome (GM) plays a significant role in IR. Individuals with T2DM exhibit distinct alterations in microbial composition compared with healthy controls (8). The GM contributes to host metabolism by extracting energy from dietary carbohydrates and generating bioactive metabolites (9). Experimental studies have shown that the GM dysbiosis can alter the production of microbial metabolites thereby influencing insulin sensitivity in the liver, adipose tissue, and muscle (10). Human observational studies further support associations between specific taxa, such as *Firmicutes* and *Bacteroidetes*, and IR (11). These findings highlight the potential of targeting specific microbial taxa and their metabolites as a therapeutic strategy for improving IR.

However, GM data are inherently high-dimensional and highly sparse, posing challenges for traditional statistical methods. Machine learning (ML) methods, by contrast, excel at handling such data structures, enabling the detection of complex, non-linear patterns within large microbiome datasets (12). ML also facilitates feature selection, offering a powerful framework for GM-based disease classification (13).

**Figure 1:**
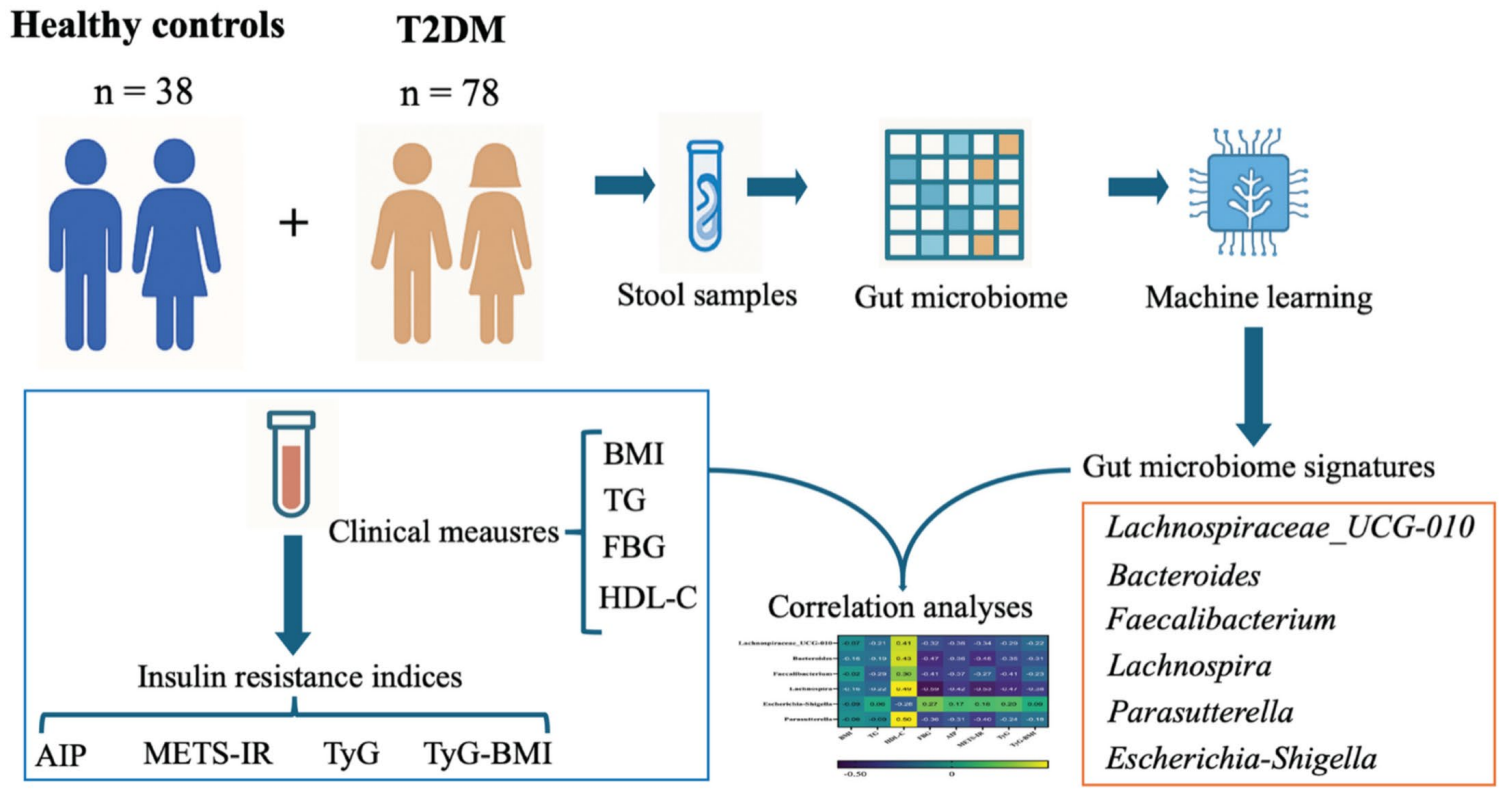
Graphical abstract. Machine-learning-based identification of gut microbiome taxa associated with insulin resistance (IR) in type 2 diabetes. A cohort of healthy controls and T2DM participants provided stool samples for gut microbiome profiling. In parallel, clinical measurements including BMI, TG, FBG, and HDL-C were collected and used to derive IR indices including AIP, METS-IR, TyG, and TyG-BMI, to capture complementary dimensions of glucose-lipid dysregulation. Microbiome features were then selected using a machine learning framework, yielding a set of IR-related microbial features. Finally, correlation analyses integrated the selected taxa with clinical and IR indices to nominate microbial features potentially relevant to IR modulation.

Therefore, we aimed to characterize the GM profiles of T2DM individuals across different IR levels, thereby refining the association between GM and IR. Specifically, we leveraged multiple IR indices to capture complementary dimensions of dysregulated glucose and lipid metabolism, and implemented a XGBoost machine learning framework to select GM features. By further correlating selected GM features with clinical and IR measures, we strengthened the biological understanding of IR-GM relationships and identified GM features with potential relevance for IR modulation. The detailed graphical abstract is shown in Figure 1.

## 2 Methods

Healthy controls were individuals undergoing routine laboratory tests at the Health Examination Center of Hospital of Chengdu University of Traditional Chinese Medicine. Patients with T2DM were recruited from the Inpatient Department of Endocrinology at the same hospital. The study received approval from the Medical Ethics Committee of Hospital of Chengdu University of Traditional Chinese Medicine (approval number 2020KL-060)^1^ and was conducted in strict adherence to the principles outlined in the Declaration of Helsinki. Informed consent was duly acquired from all participants.

### 2.1 Inclusion and exclusion criteria

Participants eligible for this study were individuals aged between 18 and 75 years. Enrollment included healthy subjects free from any ongoing diseases, as well as patients with a confirmed diagnosis of T2DM. These participants were allocated into respective groups based on their health status. The exclusion criteria were delineated as follows: (1) Individuals who have experienced acute metabolic disturbances, such as ketoacidosis, within the preceding month. (2) Presence of severe infectious diseases. (3) Concurrent liver pathologies including infections by hepatitis B or C virus, autoimmune hepatitis, primary biliary cholangitis, primary sclerosing cholangitis, drug-induced steatosis or liver injury, etc. (4) Diagnoses of gastrointestinal or hepatic malignancies. (5) History of alcoholism or substance misuse. (6) Use of antibiotics or ursodeoxycholic acid at the point of study entry. (7) Use of prebiotics or probiotics at the point of study entry. (8) Periods of pregnancy and lactation.

### 2.2 Demographic statistics

A cohort of 116 participants from Chengdu city, China, was recruited and categorized into two groups based on their health conditions: healthy controls (*n* = 38) and patients diagnosed with T2DM (*n* = 78). A Pearson chi-square test was employed to assess differences in gender distribution between the groups. Additionally, *t*-tests were utilized to evaluate disparities in age between the groups. These analyses revealed no significant differences in gender distribution or age between the groups (Table 1).

**Table 1:** Demographic summary of the control and T2DM groups.

Characteristics	Control	T2DM	*p*-value
Male	20	55	0.09
Female	18	23	
Age (years)	51.71 ± 6.66	52.90 ± 11.13	0.48

### 2.3 Gut microbiome-16S rRNA gene sequencing

Stool specimens were collected from healthy controls and T2DM patients to analyze gut microbiome profiles, which were elucidated through 16S rRNA gene sequencing. Initially, DNA was isolated from fecal samples using DNeasy PowerSoil kit, followed the assessment of amplicon quality via gel electrophoresis. The generation of the amplicon sequence variant (ASV) abundance table was performed using DADA2, employing the default parameters of set forth in QIIME2. Subsequently, all representative sequences were annotated and subjected to BLAST analysis against the Silva database (Version 138) using the q2-feature-classifier (14). The sequencing data have been deposited in the NCBI Sequence Read Archive (SRA)^2^ under the BioProject accession PRJNA1397912.

### 2.4 Blood test outcomes

After obtaining informed consent, body mass index (BMI) along with clinical measures of triglycerides (TG), high-density lipoprotein cholesterol (HDL-C), and fasting blood glucose (FBG) were extracted from the medical records of each participant. We used independent two-sample *t*-tests to compare these variables between groups. Based on these measurements, the AIP, METS-IR, TyG, and TyG-BMI were calculated for each participant using established formulas (4, 6, 7).

To examine differences across IR levels, we stratified the T2DM cohort into high and low subgroups for each index using the median as the cut-off. For AIP, the median in the T2DM group was 0.32. Participants with AIP >0.32 were assigned to the higher AIP subgroup, and those with AIP ≤0.32 to the lower AIP subgroup. The same approach was applied to other indices, with medians of 54.46 for METS-IR, 7.76 for TyG, and 199.59 for TyG-BMI.

### 2.5 Machine learning

We used subject-level leave-one-out cross-validation (LOOCV) to maximize the number of samples available for model training. In each outer LOOCV iteration, one subject was held out for testing, and the remaining subjects formed the training set. All preprocessing was performed in a leakage-free manner. Missing values in the GM matrix were imputed using a *k*-nearest neighbor (KNN) imputer fitted on the training set.

Classification was performed using an extreme gradient boosting model (XGBoost), selected for its ability to handle high-dimensional, sparse feature spaces and nonlinear feature interactions, and for its built-in L1/L2 regularization that helps reduce overfitting in small-sample settings (15). Hyperparameters were optimized within the training set using an inner cross-validation loop, and the tuned model was subsequently evaluated on the held-out subject in the outer loop. Overall model performance was computed by aggregating predictions across all LOOCV iterations.

To quantify the contribution of each feature, permutation feature importance was estimated in a cross-validation fashion by permuting one feature at a time in the held-out data and measuring the associated decrease in the chosen performance metric relative to the unpermuted baseline.

For each IR index, we constructed three separate classification tasks on the GM data: higher-index subgroup vs. controls, lower-index subgroup vs. controls, and higher vs. lower subgroups, training an XGBoost model for each comparison.

### 2.6 Correlation analysis

Correlation analyses were performed to evaluate associations among clinical measurements, IR indices and GM features selected by machine learning models. Shapiro–Wilk tests were employed to assess normality. Pearson’s correlation was used for approximately normally distributed variables with linear relationships. Otherwise, Spearman’s rank correlation was applied. All tests were two-tailed, and statistical significance was evaluated at a 95% confidence level. To account for multiple comparisons, *p*-values were adjusted using the false discovery rate (FDR) controlled by the two-stage step-up method of Benjamini, Krieger and Yekutieli, with adjusted *q* value <0.01 considered significant.

## 3 Results

### 3.1 Comparison of BMI, TG, FBG, and HDL-C between groups

**Figure 2:**
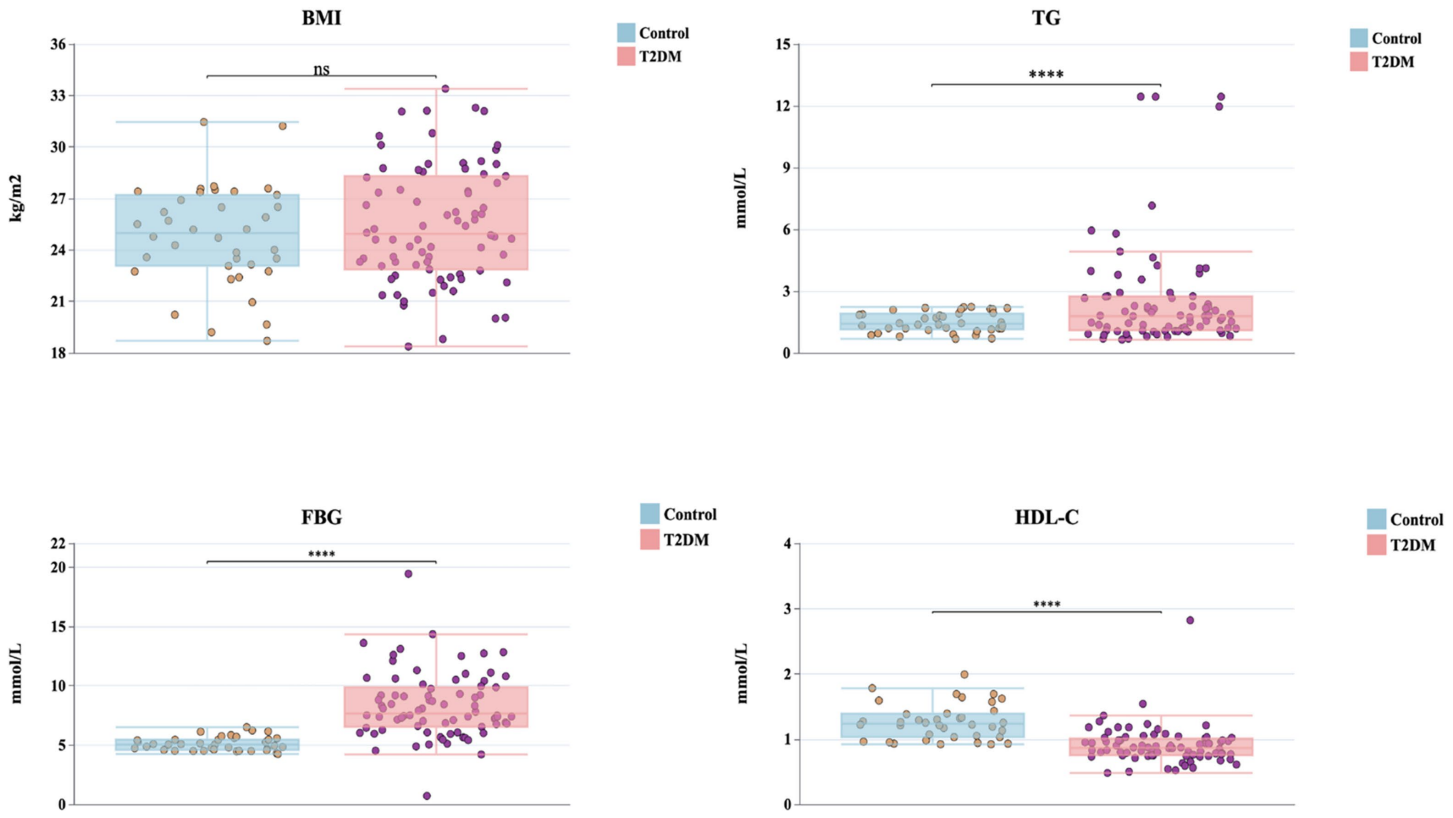
Group comparisons of clinical measures between healthy controls and individuals with T2DM. Box-and-jitter plots show BMI, TG, FBG and HDL-C in the control group (blue) and the T2DM group (pink). Each dot represents a participant. Group differences are indicated above each panel: ns denotes no significant difference, **** indicates *p* < 0.0001.

As shown in Figure 2, there was no significant difference in BMI between the T2DM and control groups (mean BMI: 25.48 vs. 24.84 kg/m^2^, *p* > 0.05). In contrast, TG, FBG, and HDL-C differed markedly between groups. Compared with controls, patients with T2DM exhibited significantly higher TG (2.60 vs. 1.49 mmol/L, *p* < 0.0001) and FBG (8.32 vs. 5.10 mmol/L, *p* < 0.0001), while HDL-C levels were significantly lower (0.91 vs. 1.27 mmol/L, *p* < 0.0001). These findings indicate that dyslipidemia and hyperglycemia, but not elevated BMI, are key distinguishing features between the groups.

### 3.2 Comparison of AIP, METS-IR, TyG, and TyG-BMI between groups

**Figure 3:**
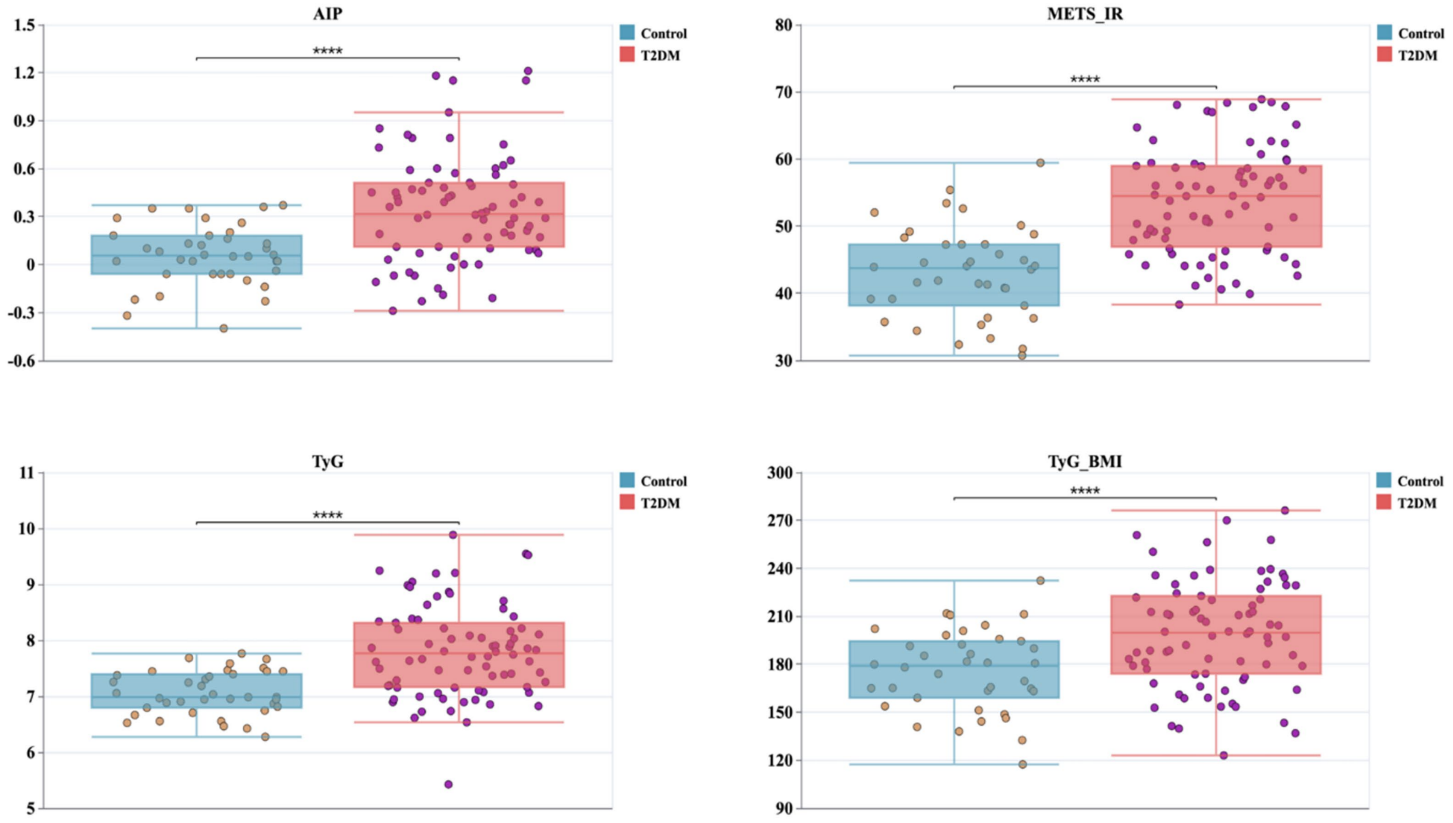
Group comparisons of insulin resistance (IR) indices between healthy controls and individuals with T2DM. Box-and-jitter plots show AIP, METS-IR, TyG and TyG-BMI in the control group (blue) and the T2DM group (pink). Each dot represents a participant. Group differences are indicated above each panel: **** indicates *p* < 0.0001.

Indices reflecting IR were consistently higher in participants with T2DM compared with healthy controls (Figure 3). The AIP was markedly elevated in the T2DM group (mean AIP 0.35 vs. 0.05 in controls), and the difference was highly significant (*p* < 0.0001). Similar patterns were seen for the METS-IR (53.89 vs. 43.04), the TyG (7.80 vs. 7.05), and the TyG-BMI (198.77 vs. 175.4), all group comparisons reached *p* < 0.0001. These findings reflect more pronounced metabolic dysregulation in T2DM group compared with healthy individuals.

### 3.3 Classification performance on GM data

**Figure 4:**
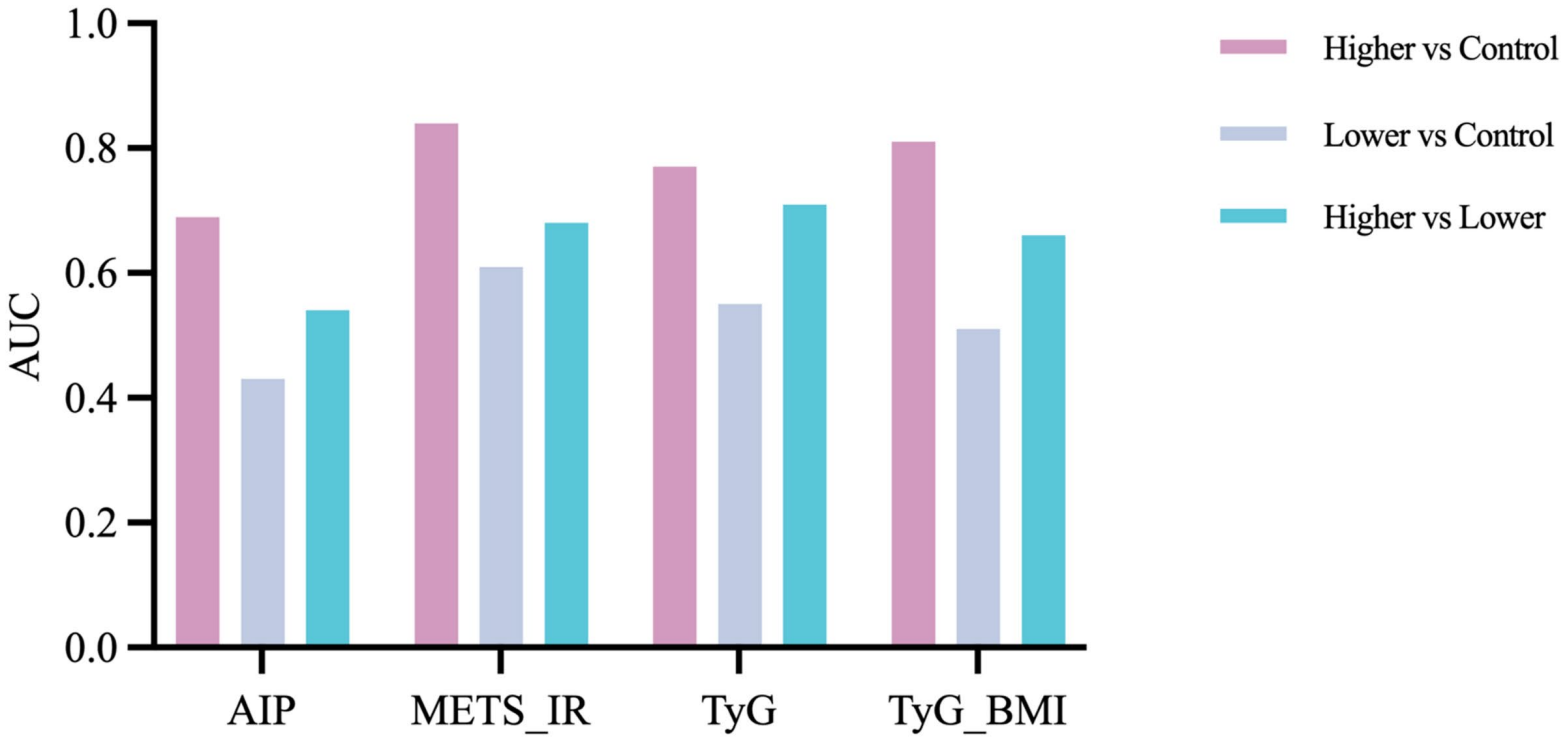
Discriminative performance of gut microbiome (GM)-based classifiers across four insulin resistance (IR) indices. Bar plots show the area under the receiver operating curve (AUC) for XGBoost classifiers constructed using GM features for AIP, METS-IR, TyG, and TyG-BMI. For each index, participants were stratified into higher and lower groups according to the index value, and classifier performance is reported for three pairwise comparisons: higher vs. control (pink), lower vs. control (light blue), and higher vs. lower (turquoise).

The XGBoost classifiers trained on GM data distinguished participants with higher IR index values from healthy controls much more effectively than other comparisons (Figure 4). For AIP, METS-IR, TyG, and TyG-BMI, the area under the ROC curve (AUC) for the higher-index vs. control models were 0.69, 0.84, 0.77 and 0.81, respectively. METS-IR achieved the strongest discrimination and AIP the weakest among these. When discriminating between higher-index and lower-index subgroups, or between lower-index and control groups, performance declined substantially. AUCs for higher vs. lower comparisons ranged from 0.54 to 0.71, and for lower vs. control comparisons from 0.43 to 0.61. Overall, these results indicate that GM signatures robustly differentiate individuals with pronounced IR from healthy controls than they separate those with less severe metabolic profiles.

### 3.4 GM feature selection based on relative contribution

**Figure 5:**
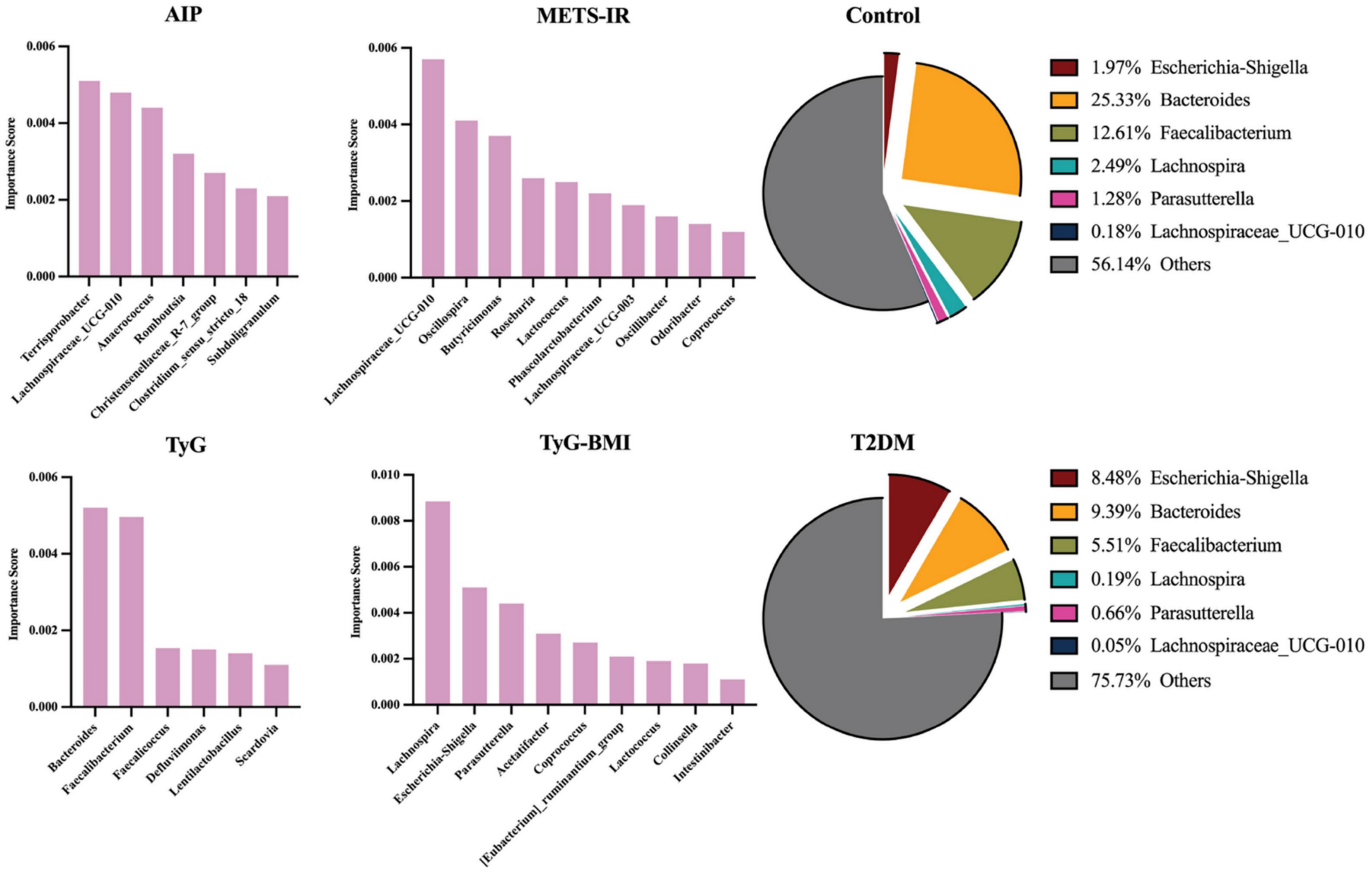
Key gut microbiome (GM) taxa contributing to classification of the higher index T2DM subgroup versus healthy controls across four insulin resistance (IR) indices. Bar plots show the feature-importance scores for the higher vs. control comparison for each index (AIP, METS-IR, TyG, and TyG-BMI). Within each panel, genera are ranked by importance (highest to lowest). The pie charts summarize the mean relative abundances of representative discriminatory genera in controls and T2DM, including Escherichia-Shigella, Bacteroides, Faecalibacterium, Lachnospira, Parasutterella, Lachnospiraceae_UCG-010, and the remaining genera are grouped as others.

Figure 5 depicts the taxa that contributed most to XGBoost models distinguishing individuals with T2DM and high IR indices from controls. Each index was driven by a distinct set of genera. In the AIP model, *Terrisporobacter* showed the greatest discriminative power, with other notable contributions from *Lachnospiraceae_UCG-010*, *Anaerococcus*, and *Romboutsia*, suggesting a broad involvement of *Firmicutes*. The METS-IR was dominated by *Lachnospiraceae_UCG-010*, followed by *Oscillospira* and *Butyricimonas*. For TyG, *Bacteroides* and *Faecalibacterium* were the most influential genera. Finally, the TyG-BMI classifier was characterized by a leading contribution from *Lachnospira*, accompanied by pathobionts including *Escherichia-Shigella* and *Parasutterella*. Consistent with these feature-importance patterns, *Escherichia-Shigella* was markedly enriched in the T2DM group compared with controls (8.48% vs. 1.97%). In contrast, the relative abundances of *Bacteroides*, *Faecalibacterium*, *Lachnospira*, *Parasutterella*, *Lachnospiraceae_UCG-010* were substantially reduced in T2DM group (25.33% vs. 9.39, 12.61% vs. 5.51, 2.49% vs. 0.19, 1.28% vs. 0.66, 0.18% vs. 0.05%, respectively).

### 3.5 Correlations among clinical measures, IR indices and GM features

Normality testing indicated that BMI, METS-IR, and TyG-BMI were approximately normally distributed, whereas all other GM features, IR indices, and clinical measures deviated from normality (Supplementary Table 1). Given the non-normal distribution of GM features, we therefore used Spearman’s rank correlation to examine associations between the selected GM features and clinical/IR measures, with FDR correction applied for multiple comparisons (Supplementary Table 2).

**Figure 6:**
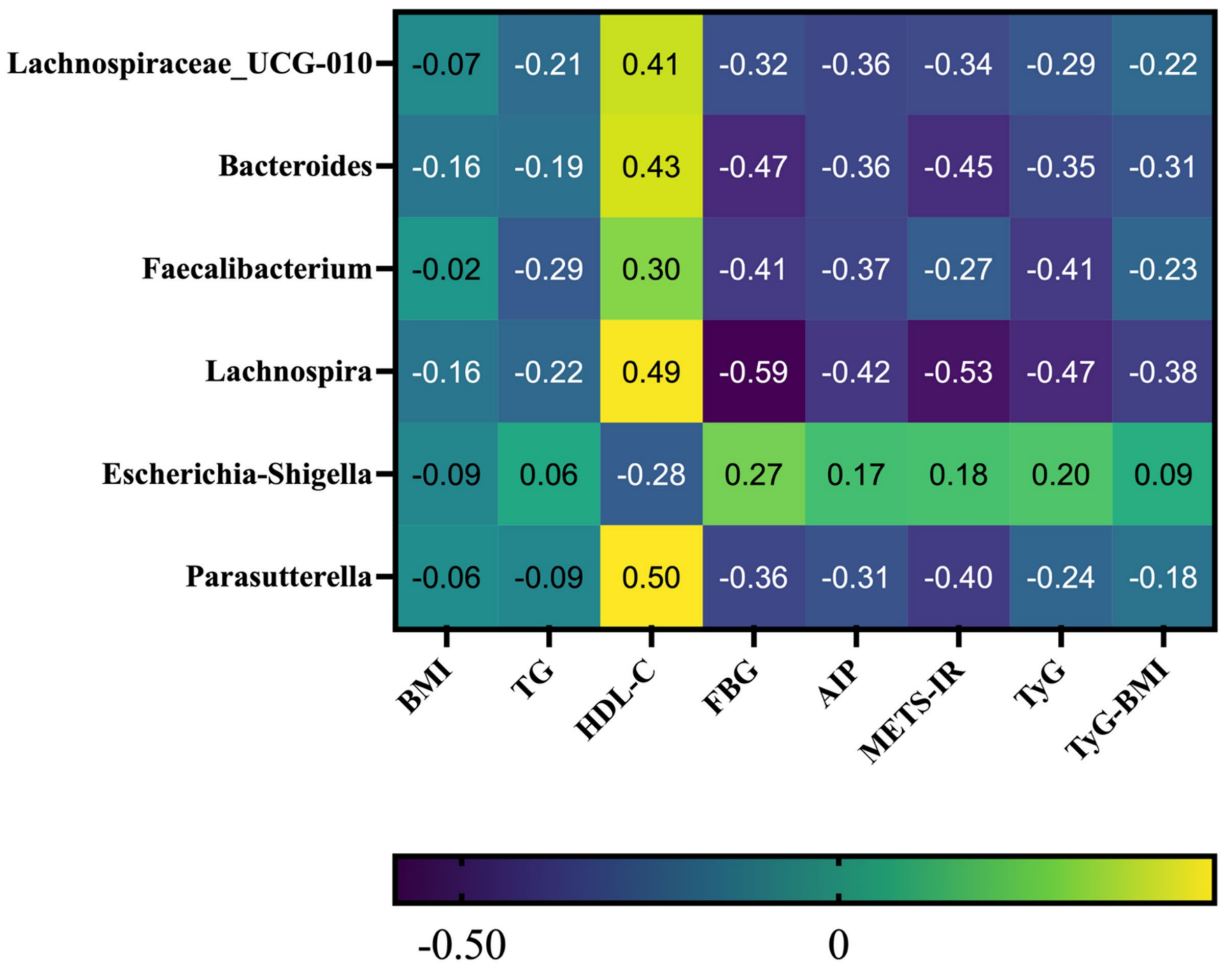
Spearman correlation heatmap between selected GM features and clinical/IR indices. Rows indicate GM features selected by the machine learning models, and columns indicate clinical/IR measures. Each cell shows the Spearman correlation coefficient. Colors represent correlation direction and magnitude, with purple indicating negative correlations and yellow indicating positive correlations.

After FDR adjustment, *Lachnospiraceae_UCG-010* was not significantly correlated with BMI, but showed negative correlations with TG, FBG, AIP, METS-IR, TyG and TyG-BMI, and a positive correlation with HDL-*C. bacteroides* was not significantly correlated with BMI or TG after FDR adjustment, but was negatively correlated with FBG, AIP, METS-IR, TyG, and TyG-BMI, and positively correlated with HDL-C. *Faecalibacterium* and *Lachnospira* were not significantly correlated with BMI after FDR adjustment, while showing negative correlations with TG, FBG, AIP, METS-IR, TyG and TyG-BMI, and positive correlations with HDL-C. For *Escherichia-Shigella*, correlations with BMI, TG, AIP, METS-IR, TyG and TyG-BMI were not significant after FDR adjustment. It showed a negative correlation with HDL-C and a positive correlation with FBG. For *Parasutterella*, correlations of BMI, TG, and TyG-BMI were not significant after FDR adjustment, whereas it showed negative correlations with FBG, AIP, METS-IR, and TyG, and a positive correlation with HDL-C (see Figure 6).

## 4 Discussion

In this study, TG, FBG, and HDL-C levels differed significantly between participants with T2DM and healthy controls. Indices of IR were also markedly higher in the T2DM group. Machine learning analysis of GM profiles demonstrated high performance in distinguishing T2DM individuals with high IR indices from healthy controls, with GM features including *Bacteroides* and *Faecalibacterium* contributing most to the XGBoost models. Correlation analyses further revealed links among clinical measures, IR indices and GM features.

Beyond elevated FBG, our results also showed that participants with T2DM exhibited abnormal lipid profiles, underscoring that the disease reflects dysregulation of both carbohydrate and lipid metabolism. IR is the core mechanism bridging these disturbances by reshaping how the liver, skeletal muscle, and adipose tissue process glucose and lipids.

In the insulin-resistant liver, gluconeogenesis remains inappropriately active and glycogen synthesis is relatively suppressed, resulting in an inadequate suppression of hepatic glucose output. Notably, this defect can coexist with preserved insulin signaling along anabolic branches. In particular, insulin upregulates SREBP1 in an mTORC1-dependent manner, and mTORC1 further enhances SREBP1 function through post-translational regulation, collectively augmenting hepatic cholesterol and fatty-acid biosynthesis and thereby promoting steatosis and hyperglyceridemia (16). Moreover, selective activation of the Akt*-*mTORC1*-*SREBP-1c axis favors intrahepatic accumulation of TG and diacylglycerols (DAGs). DAG-driven protein kinase C activation, in turn, impairs insulin receptor signaling and exacerbating hepatic IR (17).

In skeletal muscle, reduced insulin-stimulated glucose uptake limits peripheral glucose disposal, thereby increasing substrate delivery to the liver and favoring its conversion into fatty acids via *de novo* lipogenesis (18, 19). In adipose tissue, loss of insulin’s antilipolytic effect enhances adipocyte lipolysis, increasing circulating free fatty acids (FFAs) that deposit ectopically (20). Excessive lipolysis also promotes secretion of pro-inflammatory cytokines, such as IL-6 and TNF-*α*, which further impair insulin signaling and aggravate systemic insulin resistance (18).

Across these tissues, chronic nutrient excess can maintain elevated mTORC1 level, which not only promotes anabolism but also suppresses catabolic quality-control programs. Mechanistically, mTORC1 phosphorylates transcription factor EB (TFEB), retaining it in the cytoplasm and downregulating lysosomal biogenesis and autophagy gene programs. Conversely, mTORC1 inhibition activates TFEB and enhances lysosomal-autophagic processes, support proteostasis and mitochondrial clearance (16). Consistently, dietary restriction associated signaling includes mTOR inhibition along with AMPK, SIRT1, and PGC-1α, facilitating mitophagy and reducing inflammatory cytokines output, which in turn restores mitochondrial quality and improves metabolic resilience (21). However, it is important to note that overly reduced mTORC1 activity in pancreatic *β* cells may compromise insulin production and β-cell maintenance (22).

Collectively, these mechanisms highlight IR as the driver of both hyperglycemia and dyslipidemia in T2DM.

Consistently, IR indices that integrate glucose- and lipid-related measures, including AIP, METS-IR, TyG, and TyG-BMI, were significantly higher in the T2DM group. Prior studies show that higher METS-IR values often accompanied by elevated TG and reduced HDL-C, reflecting increased risk of obesity, fatty liver disease, and atherosclerosis (23–25). AIP, which captures TG/HDL-C imbalance, serves as an indicator of aggravated lipid abnormalities and heightened cardiovascular risk (26). The TyG index is a widely validated surrogate of IR, with higher values denoting impaired insulin sensitivity and disruption of glucose-lipid homeostasis (27). TyG-BMI, which incorporates adiposity, often outperforms TyG alone in detecting IR, particularly in overweight or obese populations, thereby improving early risk stratification and metabolic phenotyping in T2DM (28). Taken together, interventions targeting IR are essential not only for controlling glycemia but also for mitigating dyslipidemia and preventing cardiometabolic complications in T2DM.

Compared with medications such as metformin and GLP-1 agonists, which are effective in improving IR, GM interventions offer distinct and complementary advantages. By directly modulating host–microbe interactions, GM-targeted strategies can restore microbial balance, increase the abundance of short-chain fatty acid (SCFA)-producing bacteria, and thereby enhance endogenous GLP-1 secretion (29). These microbes not only mimic incretin-based pharmacotherapy but also reinforce the intestinal barrier and attenuate chronic low-grade inflammation (30). Clinical studies further indicate that combining probiotics with metformin reduces gastrointestinal side effects and yields greater reductions in HbA1c compared with metformin alone (31). Moreover, unlike conventional drugs that mainly target specific metabolic pathways, GM interventions adopt a multifaceted approach, simultaneously improving glucose homeostasis, lipid metabolism, and systemic inflammatory status.

In this study, we identified GM features including *Lachnospiraceae_UCG-010*, *Bacteroides*, *Faecalibacterium*, *Lachnospira*, *Parasutterella*, and *Escherichia-Shigella* that distinguished T2DM participants with high IR indices from healthy controls and were associated with clinical measures and IR indices.

*Lachnospiraceae_UCG-010*, a member of the *Lachnospiraceae* family, is a major short-chain-fatty-acid (SCFA) producer. Butyrate, in particular, modulates inflammation and stimulates glucagon-like peptide-1 (GLP-1) secretion, thereby improving glucose uptake and insulin sensitivity. Reports also show that *Lachnospiraceae_UCG-010* is correlated with oxidative stress and lipid-metabolism indices in metabolic disorders, suggesting that its abundance reflects lipid dysregulation (32). Overall, targeting *Lachnospiraceae_UCG-010* likely to support carbohydrate and lipid homeostasis through SCFA production in T2DM individuals.

*Faecalibacterium*, particularly *F. prausnitzii*, is another butyrate producer. Animal studies demonstrate that supplementation with *F. prausnitzii* significantly lowers FFA, TG, total cholesterol and the atherogenic index, while also reducing hepatic TG and cholesterol content (33). Mechanistically, this bacterium suppresses hepatic lipogenic enzymes, such as fatty-acid synthase and *β*-oxidation activities, and decreases pro-inflammatory cytokines including TNF-*α*, IL-6 and IFN-*γ*. *F. prausnitzii* is considered as an indicator of gut dysbiosis as its abundance is diminished in T2DM individuals (33). Restoring *F. prausnitzii* may improve insulin sensitivity and lipid metabolism through butyrate-mediated anti-inflammatory effects.

*Bacteroides* species produce diverse metabolites with opposing metabolic effects. On one hand, aromatic-amino-acid metabolites such as phenylacetic acid promote hepatic TG accumulation and impair insulin signaling by reducing AKT phosphorylation in hepatocytes (10). On the other, *Bacteroides* are major SCFA producers, generating acetate, propionate and butyrate (10). These SCFAs suppress hepatic lipogenesis by inhibiting lipogenic gene expression, enhance lipid oxidation via AMPK activation, increase energy expenditure, and promote adipose browning. In diabetic mice, supplementation with *Bacteroides uniformis* improved carbohydrate and lipid metabolism, lowered TG and LDL-C, down-regulated hepatic gluconeogenic and lipogenic genes, and restored bile acid signaling through the TGR5/AMPK pathway (34).

*Lachnospira* spp. produce SCFAs and contribute to flavonoid biosynthesis. Their abundance was positively correlated with pathways for flavone/flavanol biosynthesis and negatively associated with insulin and HOMA-IR (35). Studies have suggested that lower *Lachnospira* may exacerbate IR and inflammation, suggesting a protective metabolic role. Lower *Lachnospira* abundance has been associated with increased IR and inflammation, implying that this genus supports insulin sensitivity by providing butyrate and flavonoid metabolites.

In contrast, *Parasutterella* has been linked to adverse metabolic profiles. High abundance *Parasutterella* correlates with obesity and T2DM, greater carbohydrate intake, reduced serum L-cysteine (an amino acid essential for glucose homeostasis), and activation of host fatty-acid biosynthesis pathways (36). It has also been associated with hypothalamic inflammation, potentially contributing to weight gain and IR through. Importantly, weight-loss interventions have been shown to reduce *Parasutterella* abundance, suggesting its role as a modifiable marker of metabolic dysfunction.

Similarly, *Escherichia-Shigella*, a Gram-negative Enterobacteriaceae group, is consistently enriched in diabetic models. Its abundance has been positively correlated with FBG, HOMA-IR and circulating lipopolysaccharide (LPS) levels (37). Elevated *Escherichia-Shigella* exacerbates metabolic endotoxemia by increasing LPS release, which triggers systemic inflammation and worsens IR. In db/db mice, quercetin supplementation reduced *Escherichia-Shigella* abundance, restored intestinal barrier integrity, decreased circulating LPS and improved insulin sensitivity. Intermittent fasting has similar effects, reducing *Escherichia-Shigella*, improving glucose tolerance, and enhancing insulin sensitivity (38).

Overall, supplementation with SCFA-producing genera such as *Lachnospiraceae_UCG-010*, *B. uniformis*, *F. prausnitzii*, *Lachnospira* may improve IR and metabolic homeostasis by enhancing insulin signaling, promoting lipid oxidation, and reducing inflammation. Conversely, interventions such as quercetin supplementation and lifestyle modifications (e.g., weight loss, intermittent fasting) may reduce harmful genera such as *Parasutterella* and *Escherichia-Shigella*, thereby alleviating hepatic TG accumulation and systemic endotoxin-mediated inflammation.

The primary limitation of this work is the modest sample size, which may constrain generalizability and increase the risk of overfitting in ML models. In addition, the cross-sectional design captures GM composition at a single time point and therefore cannot resolve temporal trajectories, directionality, or causality in the GM-IR relationship during T2DM onset and progression. Third, residual confounding cannot be fully excluded, factors such as adiposity distribution, smoking status, dietary patterns, and medication exposure may influence both IR and GM. Fourth, the use of 16S rRNA sequencing provides limited taxonomic resolution and does not directly measure microbial functional capacity, restricting pathway-level mechanistic inference.

Future work should therefore prioritize larger, multi-center cohorts with independent external validation to improve the robustness and transportability of the identified signatures. Longitudinal designs with repeated GM sampling and comprehensive metabolic phenotyping are also needed to assess within-person dynamics and strengthen causal inference. Moreover, future studies should incorporate richer covariate collection to better control confounding and evaluate potential effect modification. Finally, mechanistic insights would be further strengthened by integrating complementary omics layers, such as shotgun metagenomics, together with targeted experimental validation.

## 5 Conclusion

In summary, this study demonstrated that the gut microbiota profiles of T2DM participants with high IR levels were significantly different from those of healthy controls. Key genera, including *Lachnospiraceae_UCG-010*, *Bacteroides*, *Faecalibacterium*, *Lachnospira*, *Parasutterella*, and *Escherichia-Shigella*, emerged as potential targets for interventions aimed at improving insulin resistance and restoring carbohydrate and lipid metabolism. Importantly, incorporating microbiota-informed strategies could translate these findings into timelier and more precise T2DM management. For instance, routine microbiome profiling may help clinicians stratify patients by dysbiosis and IR risk, anticipate inter-individual variability in response to orally administered therapies, and enable earlier, personalized adjunct interventions with longitudinal monitoring to track therapeutic response and metabolic trajectory (39).

## Data Availability

The original contributions presented in the study are publicly available. This data can be found in the NCBI Sequence Read Archive (SRA) (https://www.ncbi.nlm.nih.gov/sra) under the BioProject accession PRJNA1397912.

## Footnotes

1
http://222.212.84.254:12585/irb/query.aspx

2
https://www.ncbi.nlm.nih.gov/sra

## References

[1] American Diabetes Association Professional Practice Committee. 2. Diagnosis and classification of diabetes: standards of care in diabetes-2025. Diabetes Care. (2025) 48:S27–49. doi: 10.2337/dc25-S002, 39651986 PMC11635041

[2] Sun H Saeedi P Karuranga S Pinkepank M Ogurtsova K Duncan BB . IDF diabetes atlas: global, regional and country-level diabetes prevalence estimates for 2021 and projections for 2045. Diabetes Res Clin Pract. (2022) 183:109119. doi: 10.1016/j.diabres.2021.109119, 34879977 PMC11057359

[3] Petersen MC Shulman GI. Mechanisms of insulin action and insulin resistance. Physiol Rev. (2018) 98:2133–223. doi: 10.1152/physrev.00063.2017, 30067154 PMC6170977

[4] Qu L Fang S Lan Z Xu S Jiang J Pan Y . Association between atherogenic index of plasma and new-onset stroke in individuals with different glucose metabolism status: insights from CHARLS. Cardiovasc Diabetol. (2024) 23:215. doi: 10.1186/s12933-024-02314-y, 38907337 PMC11193183

[5] Yin B Wu Z Xia Y Xiao S Chen L Li Y. Non-linear association of atherogenic index of plasma with insulin resistance and type 2 diabetes: a cross-sectional study. Cardiovasc Diabetol. (2023) 22:157. doi: 10.1186/s12933-023-01886-5, 37386500 PMC10311747

[6] Bello-Chavolla OY Almeda-Valdes P Gomez-Velasco D Viveros-Ruiz T Cruz-Bautista I Romo-Romo A . METS-IR, a novel score to evaluate insulin sensitivity, is predictive of visceral adiposity and incident type 2 diabetes. Eur J Endocrinol. (2018) 178:533–44. doi: 10.1530/EJE-17-0883, 29535168

[7] Yan S Wang D Jia Y. Comparison of insulin resistance-associated parameters in US adults: a cross-sectional study. Hormones Athens. (2023) 22:331–41. doi: 10.1007/s42000-023-00448-4, 36972006

[8] Diamant M Blaak EE de Vos WM. Do nutrient-gut-microbiota interactions play a role in human obesity, insulin resistance and type 2 diabetes? Obes Rev. (2011) 12:272–81. doi: 10.1111/j.1467-789X.2010.00797.x, 20804522

[9] Lee CJ Sears CL Maruthur N. Gut microbiome and its role in obesity and insulin resistance. Ann N Y Acad Sci. (2020) 1461:37–52. doi: 10.1111/nyas.14107, 31087391

[10] Jang HR Lee HY. Mechanisms linking gut microbial metabolites to insulin resistance. World J Diabetes. (2021) 12:730–44. doi: 10.4239/wjd.v12.i6.730, 34168724 PMC8192250

[11] Moreno-Navarrete JM Serino M Blasco-Baque V Azalbert V Barton RH Cardellini M . Gut microbiota interacts with markers of adipose tissue browning, insulin action and plasma acetate in morbid obesity. Mol Nutr Food Res. (2018) 62:1700721. doi: 10.1002/mnfr.201700721, 29105287

[12] Hernández Medina R Kutuzova S Nielsen KN Johansen J Hansen LH Nielsen M . Machine learning and deep learning applications in microbiome research. ISME Commun. (2022) 2:98. doi: 10.1038/s43705-022-00182-9, 37938690 PMC9723725

[13] Marcos-Zambrano LJ Karaduzovic-Hadziabdic K Loncar Turukalo T Przymus P Trajkovik V Aasmets O . Applications of machine learning in human microbiome studies: a review on feature selection, biomarker identification, disease prediction and treatment. Front Microbiol. (2021) 12:634511. doi: 10.3389/fmicb.2021.634511 33737920 PMC7962872

[14] Liu G Li T Zhu X Zhang X Wang J. An independent evaluation in a CRC patient cohort of microbiome 16S rRNA sequence analysis methods: OTU clustering, DADA2, and Deblur. Front Microbiol. (2023) 14:1178744. doi: 10.3389/fmicb.2023.1178744, 37560524 PMC10408458

[15] Tarwidi D Pudjaprasetya SR Adytia D Apri M. An optimized XGBoost-based machine learning method for predicting wave run-up on a sloping beach. MethodsX. (2023) 10:102119. doi: 10.1016/j.mex.2023.102119, 37007622 PMC10064230

[16] Zhao T Fan J Abu-Zaid A Burley S Zheng XF. Nuclear mTOR signaling orchestrates transcriptional programs underlying cellular growth and metabolism. Cells. (2024) 13:781. doi: 10.3390/cells13090781, 38727317 PMC11083943

[17] Mu W Cheng XF Liu Y Lv QZ Liu GL Zhang JG . Potential nexus of non-alcoholic fatty liver disease and type 2 diabetes mellitus: insulin resistance between hepatic and peripheral tissues. Front Pharmacol. (2018) 9:1566. doi: 10.3389/fphar.2018.01566, 30692925 PMC6339917

[18] Chait A den Hartigh LJ. Adipose tissue distribution, inflammation and its metabolic consequences, including diabetes and cardiovascular disease. Front Cardiovasc Med. (2020) 7:22. doi: 10.3389/fcvm.2020.00022, 32158768 PMC7052117

[19] Wang C Wang P Chen W Bai Y. Mechanisms of *Gynostemma pentaphyllum* against non-alcoholic fibre liver disease based on network pharmacology and molecular docking. J Cell Mol Med. (2022) 26:3760–71. doi: 10.1111/jcmm.17410, 35665440 PMC9258700

[20] Morigny P Houssier M Mouisel E Langin D. Adipocyte lipolysis and insulin resistance. Biochimie. (2016) 125:259–66. doi: 10.1016/j.biochi.2015.10.024, 26542285

[21] Fan J Khanzada Z Xu Y. Mechanisms underlying muscle-related diseases and aging: insights into pathophysiology and therapeutic strategies. Muscles. (2025) 4:26. doi: 10.3390/muscles4030026, 40843913 PMC12371960

[22] Fan J Yuan Z Burley SK Libutti SK Zheng XFS. Amino acids control blood glucose levels through mTOR signaling. Eur J Cell Biol. (2022) 101:151240. doi: 10.1016/j.ejcb.2022.151240, 35623230 PMC10035058

[23] Pihlajamäki J Gylling H Miettinen TA Laakso M. Insulin resistance is associated with increased cholesterol synthesis and decreased cholesterol absorption in normoglycemic men. J Lipid Res. (2004) 45:507–12. doi: 10.1194/jlr.M300368-JLR200, 14657199

[24] Qian T Sheng X Shen P Fang Y Deng Y Zou G. Mets-IR as a predictor of cardiovascular events in the middle-aged and elderly population and mediator role of blood lipids. Front Endocrinol. (2023) 14:1224967. doi: 10.3389/fendo.2023.1224967, 37534205 PMC10393118

[25] Yoon J Jung D Lee Y Park B. The metabolic score for insulin resistance (METS-IR) as a predictor of incident ischemic heart disease: a longitudinal study among Korean without diabetes. J Pers Med. (2021) 11:742. doi: 10.3390/jpm11080742, 34442386 PMC8399912

[26] Li Y Feng Y Li S Ma Y Lin J Wan J . The atherogenic index of plasma (AIP) is a predictor for the severity of coronary artery disease. Front Cardiovasc Med. (2023) 10:1140215. doi: 10.3389/fcvm.2023.1140215, 37441702 PMC10333749

[27] Li S Xia R Gong X Wang C Liu H Dong H . Mediating effect of TyG index on the association between glucose-lipid metabolism-related dietary pattern and T2DM: a propensity score-matched analysis. BMC Endocr Disord. (2025) 25:114. doi: 10.1186/s12902-025-01892-6, 40275221 PMC12020057

[28] Zhang Y Wang R Fu X Song H. Non-insulin-based insulin resistance indexes in predicting severity for coronary artery disease. Diabetol Metab Syndr. (2022) 14:191. doi: 10.1186/s13098-022-00967-x, 36528713 PMC9759860

[29] Kyriachenko Y Falalyeyeva T Korotkyi O Molochek N Kobyliak N. Crosstalk between gut microbiota and antidiabetic drug action. World J Diabetes. (2019) 10:154–68. doi: 10.4239/wjd.v10.i3.154, 30891151 PMC6422856

[30] Lee CB Chae SU Jo SJ Jerng UM Bae SK. The relationship between the gut microbiome and metformin as a key for treating type 2 diabetes mellitus. Int J Mol Sci. (2021) 22:3566. doi: 10.3390/ijms22073566, 33808194 PMC8037857

[31] Şahin K Şahintürk Y Köker G Özçelik Köker G Bostan F Kök M . Metformin with versus without concomitant probiotic therapy in newly diagnosed patients with type 2 diabetes or prediabetes: a comparative analysis in relation to glycemic control, gastrointestinal side effects, and treatment compliance. Turk J Gastroenterol. (2022) 33:925–33. doi: 10.5152/tjg.2022.211063, 36098362 PMC9797791

[32] Yang F Chen G. Editorial: nutrients, gut microbiome, and intestinal inflammation. Front Nutr. (2022) 9:977513. doi: 10.3389/fnut.2022.977513, 35911125 PMC9335356

[33] Xuan W Ou Y Chen W Huang L Wen C Huang G . *Faecalibacterium prausnitzii* improves lipid metabolism disorder and insulin resistance in type 2 diabetic mice. Br J Biomed Sci. (2023) 80:10794. doi: 10.3389/bjbs.2023.10794 37025162 PMC10070466

[34] Zhu XX Zhao CY Meng XY Yu XY Ma LC Chen TX . *Bacteroides uniformis* ameliorates carbohydrate and lipid metabolism disorders in diabetic mice by regulating bile acid metabolism via the gut-liver axis. Pharmaceuticals. (2024) 17:1015. doi: 10.3390/ph17081015, 39204119 PMC11357665

[35] Tsai CC Chiu MH Kek HP Yang MC Su YT Liu HK . The reduced gut *Lachnospira* species is linked to liver enzyme elevation and insulin resistance in pediatric fatty liver disease. Int J Mol Sci. (2024) 25:3640. doi: 10.3390/ijms25073640, 38612453 PMC11011648

[36] Henneke L Schlicht K Andreani NA Hollstein T Demetrowitsch T Knappe C . A dietary carbohydrate—gut Parasutterella—human fatty acid biosynthesis metabolic axis in obesity and type 2 diabetes. Gut Microbes. (2022) 14:2057778. doi: 10.1080/19490976.2022.2057778, 35435797 PMC9037427

[37] Yuan M Sun T Zhang Y Guo C Wang F Yao Z . Quercetin alleviates insulin resistance and repairs intestinal barrier in db/db mice by modulating gut microbiota. Nutrients. (2024) 16:1870. doi: 10.3390/nu16121870, 38931226 PMC11206920

[38] Li Z Chen S Yin B Wei J Wang D Zhou H . Intermittent fasting regulates gut microbiota and serum metabolome profiles in middle-aged mice fed high-fat diet. Nutr Metab Lond. (2025) 22:16. doi: 10.1186/s12986-025-00904-5, 40001132 PMC11863773

[39] Khan I. Drugs and gut microbiome interactions-an emerging field of tailored medicine. BMC Pharmacol Toxicol. (2023) 24:43. doi: 10.1186/s40360-023-00684-9, 37649091 PMC10469409

